# Mesoporous Titanium Dioxide Nanoparticles—Poly(N-isopropylacrylamide) Hydrogel Prepared by Electron Beam Irradiation Inhibits the Proliferation and Migration of Oral Squamous Cell Carcinoma Cells

**DOI:** 10.3390/polym15183659

**Published:** 2023-09-05

**Authors:** Huangqin Chen, Yuzhu Hu, Chizhou Wu, Kun Liu, Rui Feng, Mingzhe Yang, Mengyao Zhao, Bin Huang, Yuesheng Li

**Affiliations:** 1 Department of Stomatology, School of Stomatology and Ophthalmology, Xianing Medical College, Hubei University of Science and Technology, Xianning 437100, China; chenhuangqin@hbust.edu.cn (H.C.);; 2 Hubei Key Laboratory of Radiation Chemistry and Functional Materials, Non-Power Nuclear Technology Collaborative Innovation Center, Hubei University of Science and Technology, Xianning 437100, China

**Keywords:** mesoporous titanium dioxide, hydrogel, electron beam radiation, astragalus polysaccharide, oral squamous cell carcinoma

## Abstract

An urgently needed approach for the treatment of oral squamous cell carcinoma (OSCC) is the development of novel drug delivery systems that offer targeted specificity and minimal toxic side effects. In this study, we developed an injectable and temperature-sensitive composite hydrogel by combining mesoporous titanium dioxide nanoparticles (MTNs) with Poly(N-isopropylacrylamide) (PNIPAAM) hydrogel to serve as carriers for the model drug Astragalus polysaccharide (APS) using electron beam irradiation. The characteristics of MTNs, including specific surface area and pore size distribution, were analyzed, and the characteristics of MTNs-APS@Hyaluronic acid (HA), such as microscopic morphology, molecular structure, crystal structure, and loading efficiency, were examined. Additionally, the swelling ratio, gel fraction, and microscopic morphology of the composite hydrogel were observed. The in vitro cumulative release curve was plotted to investigate the sustained release of APS in the composite hydrogels. The effects on the proliferation, migration, and mitochondrial membrane potential of CAL-27 cells were evaluated using MTT assay, scratch test, and JC-1 staining. The results indicated successful preparation of MTNs with a specific surface area of 147.059 m^2^/g and an average pore diameter of 3.256 nm. The composite hydrogel displayed temperature-sensitive and porous characteristics, allowing for slow release of APS. Furthermore, it effectively suppressed CAL-27 cells proliferation, migration, and induced changes in mitochondrial membrane potential. The addition of autophagy inhibitors chloroquine (CQ) and 3-methyladenine (3-MA) attenuated the migration inhibition (*p* < 0.05).

## 1. Introduction

Oral squamous cell carcinoma (OSCC) is the most common malignant tumor in the oral cavity, accounting for about 90% of oral cancers, with a high degree of malignancy and susceptibility to metastasis [1]. The mortality rate of OSCC is increasing by approximately 2% annually [2], and the 5-year survival rate is only 50–60% [3]. Conventional treatments, such as platinum-based drugs, exhibit non-specific binding properties, leading to severe systemic toxicity and significant side effects [4]. Therefore, there is an urgent need to explore novel drugs and delivery systems to enhance drug targeting and reduce toxicity on normal tissues.

The use of traditional Chinese herbal medicine in tumor treatment has garnered significant attention due to its perceived advantages of having minimal toxic and side effects. Herbal medicine has been an integral part of traditional medicine systems in many cultures for centuries and is increasingly being explored as a complementary or alternative approach to conventional cancer therapies. Astragalus polysaccharide (APS) is one of the main active components derived from the traditional Chinese herb Astragalus membranaceus [5]. It is known to possess various pharmacological properties including anti-inflammatory, immunomodulatory, and antioxidant effects [6]. APS has been reported to exhibit inhibitory effects on various tumors such as gastric cancer, lung cancer, and breast cancer [7,8]. At the animal level, APS has been shown to improve the survival rate of mice with SCC-25 xenograft models of OSCC [9]. However, APS lacks targeting ability and has low bioavailability. Therefore, there is a desire to enhance its bioactivity through the development of a drug delivery system.

Drug delivery systems are specialized approaches designed to deliver therapeutic agents to specific target sites in the body, allowing for enhanced efficacy and reduced side effects. This system plays a crucial role in optimizing drug performance by controlling the release rate and spatial distribution of drugs, thereby improving treatment outcomes. It also enhances the biodistribution of drugs, or to target them to particular cells or locations [10,11]. One promising class of materials extensively explored in drug delivery applications is mesoporous materials. Mesoporous materials are porous materials with high specific surface area and intrinsic porosity. Among them, mesoporous titanium dioxide nanoparticles (MTNs) have been widely used in drug carrier studies due to their low cytotoxicity, high drug-loading capacity, and slow drug release capability. MTNs also exhibit photocatalytic properties, generating reactive oxygen species (ROS) upon irradiation with specific wavelengths of light, which can kill tumor tissues and achieve therapeutic goals through photodynamic therapy and other biological effects [12,13,14]. Hyaluronic acid (HA) is a natural high molecular acidic mucopolysaccharide composed of N-acetyl-glucosamine and D-glucuronide. It has been shown that HA specifically recognizes CD44 receptor overexpressing tumor cells and delivers proteins, peptides, nucleic acids, and various anticancer drugs via receptor-mediated endocytosis [15]. HA modified nanoparticles offer a controlled release of doxorubicin and become an attractive alternative of drug delivery system in cancer therapy [16]. HA can also alter the biodistribution of drugs and increase drug accumulation in the tumor sites due to enhanced active targeting, resulting in improved therapeutic efficacy [17]. Poly(N-isopropylacrylamide) (PNIPAAM) is a thermosensitive polymer with a lower critical solution temperature (LCST) of approximately 32 °C, which is below the physiological temperature of the human body. Its unique thermo-responsive properties allow its solution to transition from a sol state to a more viscous gel state by increasing the temperature to 37 °C [18], making it an ideal drug delivery vehicle. This temperature-triggered gelation ensures controlled drug release, targeted delivery to specific areas like tumor sites, and enhanced drug stability during transportation and storage. Moreover, PNIPAAM-based systems offer non-invasive administration and versatile applications for a wide range of therapeutic agents, promising innovative drug delivery solutions with improved efficacy and minimized side effects. Currently, PNIPAAM-based smart hydrogels have emerged as attractive candidates for biomedical applications, including tumor treatment [19].

Therefore, in this study, we constructed thermosensitive, injectable MTNs-APS@HA-PNIPAAM composite hydrogels by electron beam irradiation using APS as the drug model, MTNs as the drug carrier, HA as the targeting ligand, and PNIPAAM as polymers. We characterized the specific surface area, pore size, microscopic morphology, molecular structure, and crystal structure of MTNs and MTNs-APS@HA. We also characterized the micromorphology, swelling ratio, and gel fraction of the composite hydrogels. Additionally, we evaluated the loading efficiency of MTNs and the release profile of APS in composite hydrogels. We also employed MTT assay, cell scratch test, and JC-1 staining to assess the impact of the hydrogel on the proliferation and migration capability of CAL-27 cells, as well as the mitochondrial membrane potential.

Electron beam irradiation technology is a process that utilizes high-energy electron beams to modify and treat materials. When applied to hydrogel preparation, this technique achieves rapid and efficient cross-linking of polymer chains, leading to the formation of a stable and robust three-dimensional network within the hydrogel. Moreover, with no need for chemical initiators and high levels of sterilization, electron beam irradiation offers an ideal choice for a wide range of biomedical applications. The aim of this study is to fabricate MTNs-APS@HA-PNIPAAM hydrogels using electron beam irradiation and subsequently characterize them. These hydrogels are intended to be used for investigating their effects on the proliferation and migration of CAL-27 cells, which serve as a representative oral squamous cell carcinoma (OSCC) cell line. Furthermore, the study seeks to explore the preliminary mechanisms underlying the impact of MTNs-APS@HA-PNIPAAM hydrogels on CAL-27 cell behavior. This study will enrich and complement the application of MTNs, hydrogels, and electron beam irradiation technology in the active targeting of natural drugs to tumor cells with great scientific significance. This study also employs a network pharmacology method to predict and search the prognosis-related hub genes for targeted treatment of OSCC, and validates them by molecular docking, which will further provide data support for the development of astragalus-related pharmaceutical products.

## 2. Materials and Methods

### 2.1. Preparation of MTNs-APS@HA-PNIPAAM Hydrogels

To prepare the MTNs nanoparticles, 30% titanium oxide sulfate and tert-butanol are mixed in a ratio of 1:20. After 15 min of ultrasound treatment, the mixture is transferred to a Teflon-lined hydrothermal autoclave and kept at 115 °C for 12 h. Afterward, the sample is removed and washed twice with ethanol and distilled water, followed by drying at 70 °C for 60 min. The dried sample is then calcined at 380 °C in a muffle furnace, resulting in the formation of MTNs nanoparticles.

To prepare MTNs-APS, an appropriate amount of MTNs and APS is dispersed in 1 mL of water, and then subjected to 10 min of ultrasonic dispersion. After centrifugation, the precipitation is freeze-dried to obtain MTNs-APS.

Next, MTNs-APS is dispersed in 10 mL of Tris buffer, and hyaluronic acid (HA) is added. The mixture is stirred in the dark for 8 h. After centrifugation (12,000 rpm, 10 min), the precipitate is collected and washed three times to obtain MTNs-APS@HA. If MTNs are directly dispersed in a Tris solution with HA, MTNs@HA is prepared.

To prepare the final composite hydrogel, MTNs-APS@HA is mixed with pre-irradiated 10% PNIPAAM and subjected to ultrasound mixing. The mixture is then irradiated with a 1 MeV electron beam (25 kGy, 5 kGy/pass). The control group is MTNs@HA-PNIPAAM hydrogels, which is prepared by MTNs@HA and PNIPAAM in the same way.

### 2.2. Characterization of MTNs-APS@HA-PNIPAAM Hydrogels

#### 2.2.1. Characterization of MTNs

The crystal structure and molecular structure were characterized using X-ray diffraction (XRD) (DMAX-D8X, Rigaku, Tokyo, Japan) and Fourier transform infrared spectroscopy (FTIR) (NICOLET 5700 spectrometer, Thermo Fisher Nicolet, Madison, WI, USA). XRD analysis provides information about the crystal lattice and phase identification of the MTNs, while FTIR analysis helps identify the functional groups present in the material.

The microscopic morphology of MTNs was observed by transmission electron microscopy (TEM). TEM allows for high-resolution imaging of the sample, providing insights into the morphology and internal structure of MTNs. Energy dispersive spectrum (EDS) analysis was used to examine the elemental composition and distribution in a sample. It involves bombarding the sample with high-energy particles or photons, causing the atoms in the sample to emit characteristic energy levels or wavelengths. These emitted energies are then detected and analyzed to identify the elemental composition and determine their relative abundance.

The specific surface area and pore size distribution were examined by N_2_ adsorption method. This technique, such as the Brunauer–Emmett–Teller (BET) method, measures the amount of gas adsorbed at different pressures to calculate the surface area and pore characteristics of the material.

#### 2.2.2. Characterization of Composite Hydrogels

The hydrogels were freeze-dried and the microscopic morphology of the cross-section was observed by scanning electron microscopy (SEM) after gold spraying on the surface. The swelling ratio (SR) provides a quantitative measure of the hydrogel’s responsiveness to temperature changes and its ability to absorb and retain water. To perform this experiment, hydrogel samples with similar shape and size were weighed and immersed in deionized water for different time intervals. After removing the samples from the water, any surface moisture was gently removed using filter paper, and the samples were weighed again. The swelling ratio (SR) was calculated using the following formula:

(1) $$SR(\%)=\frac{W_{s}-{\;W}_{d}}{W_{d}}\times 100\%$$

where *Ws* is the weight of the hydrogel at equilibrium swelling in deionized water and *W_d_* is the initial weight of the hydrogel before immersion in deionized water.

The gel fraction (FG) represents the percentage of the hydrogel’s weight that remains after the swelling and drying process. It provides an indication of the crosslinking efficiency and structural integrity of the hydrogel. To calculate the gel fraction, the hydrogels with similar shapes and sizes were thoroughly dried in a 60 °C oven and subsequently immersed in a 80 °C constant temperature water bath for 24 h. Afterward, the hydrogels are dried and weighed again. The gel fraction is calculated using the following formula:

(2) $$FG(\%)=\frac{W_{e}}{W_{d}}\times 100\%$$

where *W_d_* is the weight of the dried hydrogel and *W_e_* is the weight of the hydrogel at equilibrium swelling in distilled water at 80 °C.

### 2.3. Drug Delivery Related Properties

#### 2.3.1. Loading Efficiency

The drug loading efficiency (LE) represents the percentage of the drug that is successfully entrapped within carriers. To determine the drug loading efficiency of MTNs-APS nanoparticles, the supernatant from centrifuged MTNs loaded with the drug APS is analyzed using a UV-visible spectrophotometer. The absorbance of the supernatant is measured and compared to a pre-established standard curve of APS to calculate the concentration of APS in the solution. The drug loading efficiency is then calculated using the following formula:

(3) $$LE(\%)=\frac{Input\;APS\;quality-APS\;mass\;in\;the\;solution}{MTNs-APS\;nanomass}\times 100\%$$

#### 2.3.2. Drug Release In Vitro

To evaluate the drug release, the hydrogel was placed in a 2 mL centrifuge tube and 1 mL of phosphate-buffered saline (PBS) is added. The tube is then placed on a shaker at 37 °C. At predetermined time points (3, 6, 12, 24, 48, 72, 96, 120, 144, 168, 240, and 336 h), 0.5 mL of the release medium is collected and replaced with an equal volume of fresh PBS. The absorbance at 490 nm is measured, and the cumulative release curve of APS is calculated and plotted.

### 2.4. Biological Properties of MTNs-APS@HA-PNIPAAM Hydrogels

#### 2.4.1. Effect on the Proliferation of CAL-27 Cells (MTT)

To perform the cell viability assay, CAL-27 were seeded in 96-well cell culture plates and incubated for 12 h until they adhere to the plate. After cell adhesion, 100 μL of culture medium containing hydrogels with different concentrations of APS was added to each well. After the 24 h incubation, 100 μL of MTT solution was added to each well and the plate was incubated for an additional 4 h. After incubation, 150 μL of DMSO was added to each well and the plate was shaken for 10 min to dissolve the purple formazan crystals formed by viable cells. The absorbance at 490 was then measured using a microplate reader.

#### 2.4.2. Effect on the Migration of CAL-27 Cells (Scratch Test)

CAL-27 cells were seeded in 6-well plates and incubated overnight. Then, a straight scratch was made in the cell monolayer using a fine scratch tool or a scratcher. Care should be taken to maintain consistency in the scratch tool or knife to ensure similar scratch shape and width. Next, immediately after creating the scratch, a photograph was taken using a cell microscope as the 0 h time point. Subsequently, the culture medium containing the composite hydrogels was added to each well (Control group: MTNs@HA-PNIPAAM hydrogels), and the cells were further incubated under constant conditions (such as 37 °C, 5% CO_2_) for 24 h. At the end of the incubation period, the same area was photographed again using a cell microscope, and the width of the scratch was measured. The change in scratch width can be calculated and analyzed using image processing software or manual measurements. By comparing the scratch widths between the hydrogel-treated group and the control group, the impact of the hydrogel on CAL-27 cell migration and scratch healing can be evaluated.

#### 2.4.3. Effect on Mitochondrial Membrane Potential of CAL-27 Cells (JC-1 Staining)

After seeding CAL-27 cells in the wells of the plate and allowing them to adhere overnight, the hydrogel was added to the wells for co-culture for 24 h. Following the co-culture period, the cells were stained with JC-1 staining working solution and incubated for 20 min. After the incubation, the cells were washed twice with JC-1 staining buffer to remove any unbound dye. The stained cells were then observed and photographed under a fluorescence inverted microscope. By examining the fluorescence patterns and intensities, the mitochondrial membrane potential can be assessed based on the aggregation or dispersion of JC-1 dye in the cells.

### 2.5. Mechanism of APS for OSCC

#### 2.5.1. Network Pharmacology Analysis

After downloading clinical and RNAseq samples of OSCC, including 504 cancer tissue samples and 44 adjacent normal tissue samples from the TCGA database (https://portal.gdc.cancer.gov/) (accessed on 20 June 2023), APS target genes specific to OSCC were predicted and subjected to gene ontology (GO) and Kyoto encyclopedia of genes and genomes (KEGG) pathway enrichment analysis. The protein–protein interaction (PPI) network was constructed and then hub genes associated with APS and OSCC prognosis were screened. One of the selected hub genes was subjected to molecular docking studies with APS. This analysis helps explore the binding interactions and potential molecular mechanisms underlying the therapeutic effects of APS in OSCC.

#### 2.5.2. In Vitro Analysis

The autophagy inhibitors, chloroquine (CQ) and 3-methyladenine (3-MA), were used to investigate the mechanism of hydrogels on CAL-27 cells. After CAL-27 cells were seeded in 6-well plates overnight, the autophagy inhibitors were pre-incubated for 1 h and then scratch test and JC-1 staining were performed.

## 3. Results

### 3.1. Characterization

#### 3.1.1. Characterization of MTNs and MTNs-APS@HA

**Figure 1:**
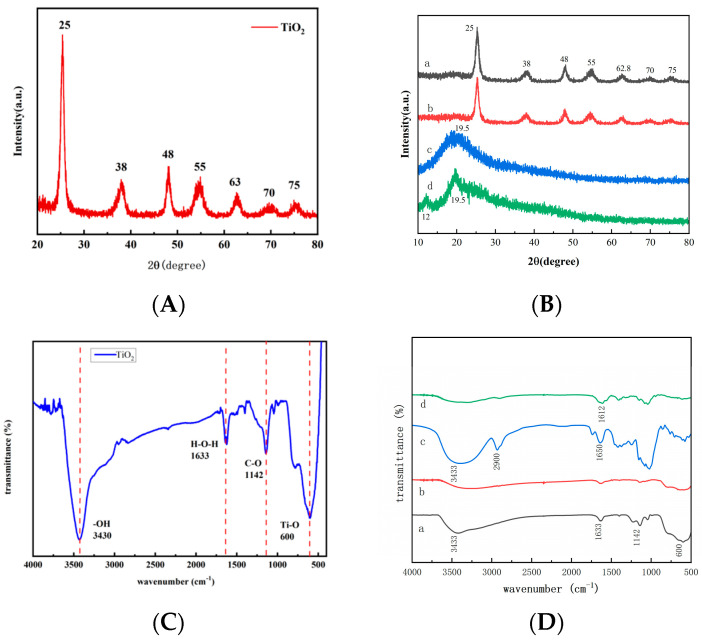
X−ray diffraction patterns (**A**,**B**) and FT−IR absorption spectrum (**C**,**D**) of MTNs and MTNs-APS@HA. a: MTNs; b: MTNs-APS@HA; c: APS; d: HA.

The X-ray diffraction (XRD) analysis of MTNs revealed characteristic peaks and patterns indicative of its crystalline structure. The obtained XRD spectrum displayed distinct diffraction peaks at specific angles, corresponding to the crystallographic planes present in the material (Figure 1A). The main peak observed at a 2 θ angle of around 25° indicates the presence of the anatase phase of MTNs. This peak is broadened, suggesting the existence of a mesoporous structure within the material. Additionally, the anatase phase is confirmed by the presence of other diffraction peaks at approximately 38°, 48°, and 55°. Furthermore, the absence of characteristic diffraction peaks associated with the rutile phase of MTNs suggests that the material primarily consists of the anatase phase. This is in line with the desired properties of mesoporous MTNs, as the anatase phase typically exhibits higher surface area and photocatalytic activity compared to the rutile phase. Figure 1B presents the XRD spectra of MTNs, MTNs-APS@HA, APS, and HA, revealing that the loading of APS and encapsulation of HA did not alter the crystal structure of MTNs.

The Fourier transform infrared spectroscopy (FT−IR) analysis of MTNs provided valuable insights into its chemical composition and functional groups. The obtained FT−IR spectrum displayed characteristic absorption bands, which allowed for the identification of various molecular vibrations within the material. In the FT−IR spectrum (Figure 1B), a broad absorption band in the range of 3200–3600 cm^−1^ suggests the presence of hydroxyl (-OH) groups, indicating the presence of surface hydroxyl groups on MTNs. This is consistent with the typical behavior of TiO_2_ materials, where hydroxyl groups are commonly found on the surface due to its strong affinity for water molecules. Additionally, a strong absorption band observed around 1633 cm^−1^ corresponds to the stretching vibrations of adsorbed water molecules. This indicates the presence of water molecules trapped within the mesoporous structure, which can contribute to the material’s unique properties and potential applications. Furthermore, the appearance of absorption bands in the range of 500–1000 cm^−1^ can be attributed to the stretching vibrations of the Ti-O bonds in the TiO_2_ lattice. These bands provide evidence of the presence of the TiO_2_ structure within the mesoporous material. Figure 1D displayed the infrared spectra of MTNs, MTNs-APS@HA, APS, and HA, revealing significant changes in chemical composition and functional groups in MTNs-APS@HA compared to MTNs. This indicated the successful loading of APS and HA.

**Figure 2:**
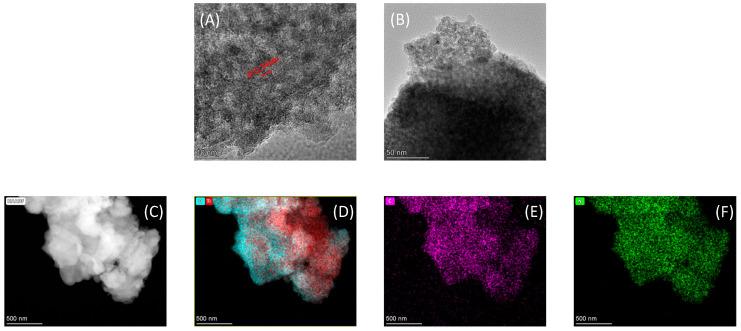
TEM and EDS analysis of MTNs and MTNs-APS@HA. (**A**) TEM of MTNs; (**B**) TEM of MTNs-APS@HA; (**C**–**F**) EDS of MTNs-APS@HA.

The transmission electron microscopy (TEM) analysis provided detailed information about morphological characteristics at the nanoscale. The obtained TEM images revealed that the synthesized MTNs exhibit a well-defined morphology characterized by a distinct worm-like mesoporous structure (Figure 2A). From Figure 2A, it was evident that the lattice fringe spacing of the MTNs crystals was approximately 0.34 nm, corresponding to the (101) crystal plane of anatase-type TiO_2_. This indicated that MTNs possessed highly crystalline mesoporous walls. Figure 2B demonstrates the alternating distribution of worm-like structures and amorphous crystal structures, indicating the successful incorporation of APS and HA. This finding was consistent with the results obtained from the infrared spectra. The energy dispersive spectrum (EDS) analysis of sample revealed distinct peaks corresponding to the presence of titanium (Ti) and oxygen (O) elements. This analysis confirmed the homogeneous distribution of Ti and O throughout the mesoporous structure of the titanium dioxide, suggesting a consistent composition across the sample (Figure 2D). Furthermore, Figure 2E,F demonstrated the presence of uniform carbon and nitrogen elements in the prepared MTNs, providing further evidence of the successful loading of APS and HA.

**Figure 3:**
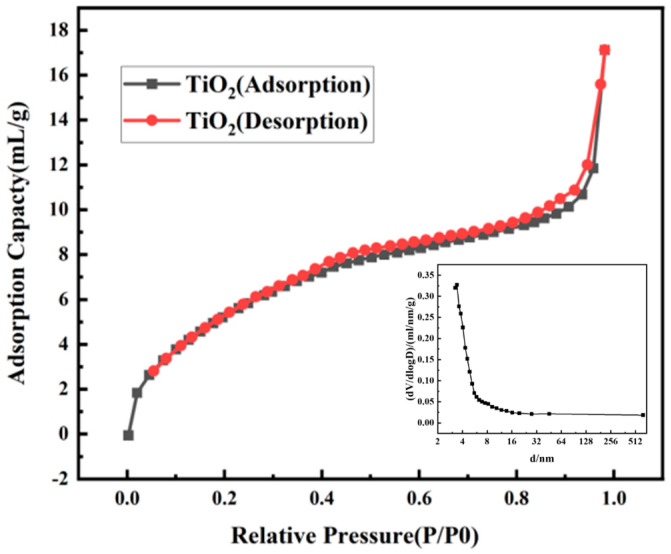
The nitrogen adsorption−desorption isotherm curve of MTNs.

The specific surface area and pore diameter distribution were determined by analyzing the nitrogen adsorption-desorption isotherm curve. Figure 3 represented the adsorption-desorption isotherms of the prepared samples, exhibiting a typical IV-type isotherm. These characteristics indicate the presence of mesopores and confirm the existence of a well-developed porous structure within the titanium dioxide microspheres. The specific surface area of the sample, calculated using the BET method, was determined to be 147.059 m^2^/g. The average pore size, obtained from the analysis, was found to be 3.256 nm. The high specific surface area means there is a large number of surface active sites available for chemical reactions, such as adsorption, catalysis, and photocatalysis. The pore size of MTNs is a crucial factor influencing the drug release rate. When the pore size of MTNs falls within a certain range, the drug release rate shows a significant increase with larger pore sizes. In drug delivery systems, a suitable pore size allows for the controlled release of drugs at specific rates, improving therapeutic efficacy and reducing side effects.

#### 3.1.2. Characterization of Composite Hydrogel

**Figure 4:**
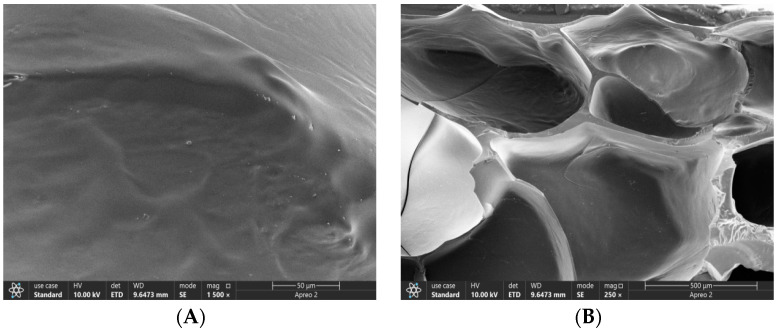
The microscopic morphology of composite hydrogel under SEM. (**A**) Surface; (**B**) section.

The scanning electron microscopy (SEM) images (Figure 4) reveal the morphological characteristics of the composite hydrogel. The surface appears relatively smooth, while the interior exhibits a three-dimensional network structure with interconnected pores. This hydrogel possesses a highly porous structure, providing a pathway for the diffusion of liquids and allowing for the exchange of nutrients and waste materials.

**Figure 5:**
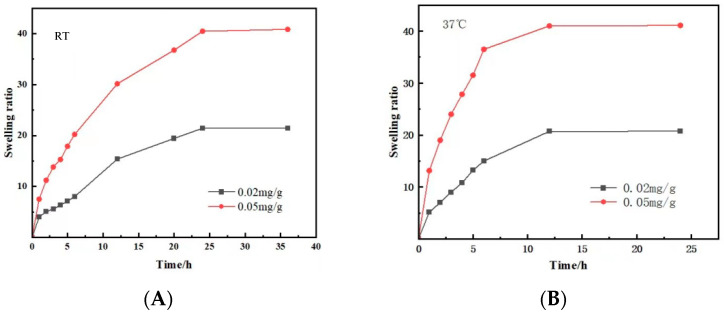
The swelling rate of composite hydrogel with different concentrations (0.02 mg/g and 0.05 mg/g) of MTNs. (**A**) Room temperature (RT); (**B**) 37 °C.

The results of the swelling ratio (SR) (Figure 5) showed that at room temperature, the composite hydrogel loaded with 0.02 mg/g MTNs exhibited a swelling ratio of about 20% at 24 h, indicating a moderate degree of water uptake. The hydrogel absorbed water and expanded to a certain extent while maintaining its structural integrity. In comparison, the composite hydrogel loaded with 0.05 mg/g MTNs displayed a higher swelling ratio of 40%. This indicates a greater water uptake capacity, resulting in more pronounced expansion of the hydrogel. When the temperature increased to 37 degrees Celsius, the equilibrium swelling time of composite hydrogel reduced from 24 h to 12 h. The higher temperature facilitated the diffusion of water molecules into the hydrogel matrix, allowing it to absorb water and swell more quickly. Overall, the results demonstrate that both composite hydrogels loaded with different concentrations of MTNs exhibited temperature-dependent swelling behavior, with higher loading concentrations and elevated temperatures promoting greater water absorption and expansion of the hydrogel. This temperature-dependent swelling behavior observed in the hydrogels suggests that they have the potential to act as responsive drug carriers, where changes in temperature can trigger controlled drug release. This could be particularly beneficial in applications where localized drug release is desired, such as in targeted therapy for tumors or inflammation sites.

The gel fraction (FG) represents the proportion of the hydrogel that forms a stable, crosslinked network structure after the synthesis process. A higher gel fraction indicates a greater extent of crosslinking and a more robust and stable hydrogel structure. In this case, the composite hydrogel loaded with 0.05 mg/g MTNs displayed a slightly higher gel fraction of 88.46% compared to the hydrogel loaded with 0.02 mg/g, which had a gel fraction of 86.70%. This suggests that the higher loading concentration of MTNs contributed to a slightly higher degree of crosslinking and improved the overall gel formation of the hydrogel. A more crosslinked network structure can result in a controlled and sustained drug release profile, preventing burst release and maintaining a steady release rate over time. This controlled drug release is particularly advantageous for achieving desired therapeutic effects and reducing potential side effects associated with rapid drug release.

### 3.2. Drug Delivery Related Properties

Loading efficiency (LE) refers to the effectiveness of a carrier material in capturing and retaining a specific drug or substance. In this experiment, the loading efficiency of MTNs for APS is determined to be 72.9% based on the analysis using the APS standard curve, which suggests that the MTNs particles have a high capability to efficiently load and retain APS.

**Figure 6:**
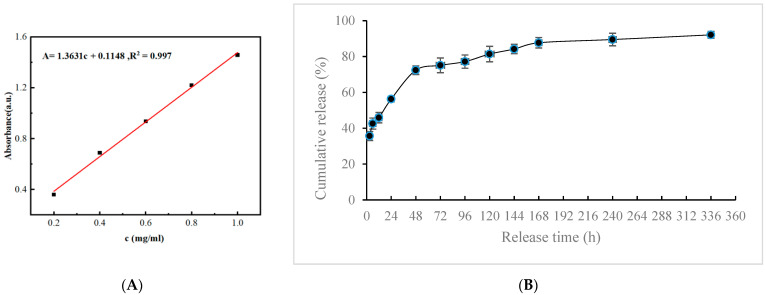
Standard curve (**A**) of APS and cumulative release (**B**) at 37 °C at predetermined time points (3, 6, 12, 24, 48, 72, 96, 120, 144, 168, 240, and 336 h).

The drug release profile from the composite hydrogel is shown in Figure 6. Within the first 3 h, approximately (35.7 ± 2.5)% of the initial drug load was released, accompanied by a minor burst release phenomenon. Subsequently, a sustained and slow drug release is observed. The cumulative drug release increases from (56.3 ± 1.5)% at 24 h (1 day) to (87.7 ± 2.9)% at 168 h (7 days). After 7 days, only a negligible amount of drug is released from the composite hydrogel. The remaining drug within the composite hydrogel accounts for 6–10% of the initial drug load.

### 3.3. Effects of MTNs-APS@HA-PNIPAAM Hydrogels on CAL-27

#### 3.3.1. Effect on Proliferation of CAL-27 Cells (MTT)

**Figure 7:**
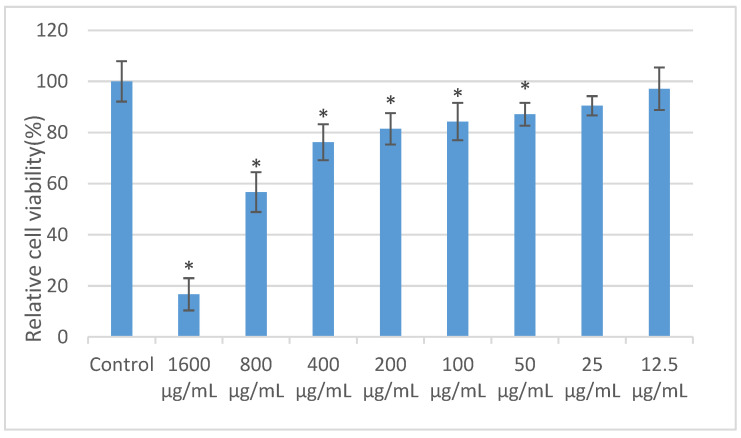
Effect of MTNs-APS@HA-PNIPAAM hydrogels on the cell viability of CAL-27. MTNs-APS@HA-PNIPAAM hydrogels with different concentrations of APS were co-cultured with CAL-27 cells for 24 h, and cell viability was detected by MTT assay. *: *p* < 0.05 compared with control group.

The effect of MTNs-APS@HA-PNIPAAM hydrogel with different concentrations of APS on the proliferation of CAL-27 cells was assessed using the MTT assay. The results (Figure 7) demonstrate that as the concentration of APS increases, the proliferation activity of CAL-27 cells decreases, indicating a concentration-dependent inhibition of cell proliferation by the hydrogel (*p* < 0.05). Compared to the control group, when the concentration of APS in the hydrogel exceeds 400 μg/mL, the proliferation rate of CAL-27 cells significantly decreases and remains below 60%. At this concentration, APS exhibits strong cytotoxicity towards CAL-27 cells. However, when the concentration of APS in the hydrogel is below 400 μg/mL, APS shows low or no toxicity towards CAL-27 cells. Therefore, for subsequent experiments, hydrogels with APS concentrations of 25, 50, 100, 200, and 400 μg/mL were selected for further investigation.

#### 3.3.2. Effect on Migration of CAL-27 Cells (Scratch Test)

**Figure 8:**
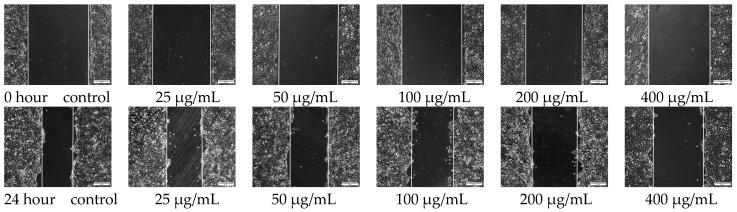
Effect of MTNs-APS@HA-PNIPAAM hydrogels with different concentrations of APS on migration of CAL-27 cell scratching assay was performed. The area of scratch was observed under a microscope (×10). 0 h: before the addition of composite hydrogel, 24 h: after adding the composite hydrogel for 24 h.

The cell scratch test is a simple method to determine cells migration and repair capacity, which simulates the migration process of cells during wound healing in vivo. When the cells grow to form a confluent monolayer and create a blank area (referred to as a scratch), the cells at the edge of the scratch gradually migrate into the blank area to close the “wound”. Images are captured at regular intervals during the initiation and cell migration process, and the speed of cell migration is quantitatively compared by measuring the width of scratch in the images [20]. In this study, the cell scratch assay was performed to evaluate the effect of MTNs-APS@HA-PNIPAAM hydrogels on the migratory ability of CAL-27 cells. After 24 h of incubation, the results showed that compared to 0 h, migration was observed in all groups, but the migration capability of CAL-27 cells weakened with increasing concentration of APS, showing a concentration-dependent effect (*p* < 0.05). As shown in Figure 8, the scratch area was calculated using ImageJ software (ImageJ 1.8.0), and at 0 h, the scratch areas in all groups were approximately 1,048,034 ± 25,166 pixels^2^, with no significant difference. After co-culturing with hydrogels with different concentrations of APS for 24 h, a reduction in scratch area was observed in all groups. The largest area was observed in the 400 μg/mL concentration group (906,130 ± 24,058 pixels^2^), while the smallest area was observed in the control group (572,674 ± 39,452 pixels^2^). The cell migration rate in the hydrogel groups after 24 h of incubation was lower than that in the control group, indicating that the hydrogel exerted an inhibitory effect on CAL-27 cell migration, and this effect was concentration dependent.

#### 3.3.3. Effect on Mitochondrial Membrane Potential of CAL-27 Cells (JC-1 Staining)

**Figure 9:**
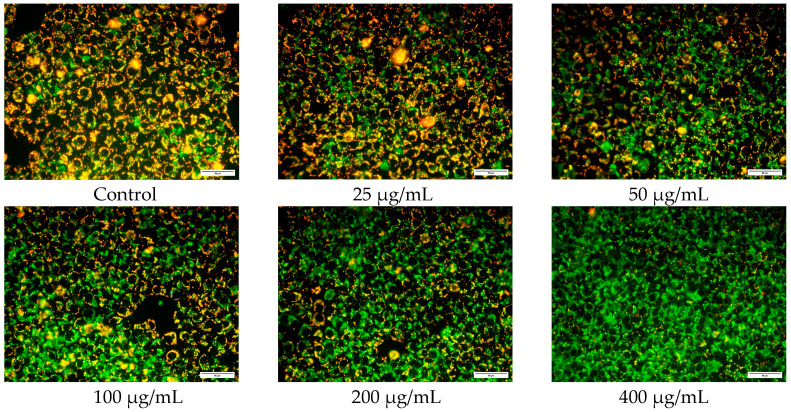
Effect of MTNs-APS@HA-PNIPAAM hydrogels with different concentrations of APS on mitochondrial membrane potential of CAL-27. JC-1 staining was performed. The fluorescence was observed under a microscope (×40).

JC-1 is a novel cationic anthocyanin dye that accumulates in the mitochondria. At low concentrations, this dye exists as monomers, emitting green fluorescence similar to fluorescein. At higher concentrations, it forms J-aggregates, resulting in red fluorescence with a broad excitation spectrum and a maximum emission wavelength of approximately 590 nm [21]. These features make JC-1 a sensitive marker for mitochondrial membrane potential ΔΨ, which can be used to assess changes in mitochondrial membrane potential [22]. Loss of mitochondrial membrane potential usually occurs during the process of drug-induced tumor cell death [23]. Therefore, in this study, JC-1 staining method was employed to evaluate the effect of the hydrogels with different concentrations of APS on the mitochondrial membrane potential of CAL-27 cells. As shown in Figure 9, the control group exhibited more red fluorescence and less green fluorescence, while the 400 μg/mL group showed the opposite pattern. Additionally, there were no significant differences between the 25 μg/mL group and the control group. However, as the concentration of APS in the hydrogel increased from 50 μg/mL to 400 μg/mL, the green fluorescence gradually increased, while the red fluorescence decreased, demonstrating a concentration-dependent effect. These results indicate that hydrogel induces cell apoptosis in CAL-27 cells, and its mechanism may be related to autophagy.

### 3.4. Mechanism of APS for OSCC

#### 3.4.1. Network Pharmacological Analysis

Due to the multiple targets and pharmacological effects of herbal medicines, traditional therapeutic methods cannot fully elucidate the mechanisms in the treatment of diseases. Network pharmacology is a multidisciplinary field that integrates principles from pharmacology, systems biology, and network analysis to study the interactions between drugs and biological systems at a network level. It aims to elucidate the complex relationships between drug targets, signaling pathways, and disease-related proteins, offering a holistic understanding of drug action and therapeutic effects. Its ability to uncover multi-target interactions and holistic therapeutic effects makes it a promising approach to study the mechanism of herbal remedies from network pharmacology [24].

In this study, a total of 2234 differential genes of OSCC were found, including 1624 upregulated genes and 610 downregulated genes (Figure S1). One hundred potential therapeutic targets of APS were obtained from SwissTarget Prediction, and only nineteen common genes remained after intersection with OSCC differential genes. We performed GO and KEGG pathways enrichment analyses for the common genes and results showed that the mechanism was mainly associated with cell autophagy and PI3K-Akt signaling pathway (Figure S2). COX regression analysis of the common genes showed that AURKA was the hub gene related to OSCC prognosis.

AURKA is a serine-threonine kinase that contributes to the regulation of mitosis in cell cycle progression. The AURKA-directed small molecule inhibitor VX-680 disrupts the bipolar spindle structure, thereby inhibiting cell growth and inducing apoptosis [25]. Studies have shown that AURKA is an important tumor regulator, which is upregulated in OSCC cell lines and tumor specimens from patients. OSCC patients with high expression of AURKA exhibited a significant decreased overall survival rate [26]. Knockdown of AURKA suppressed proliferation, migration, and invasion of OSCC cells, demonstrating that AURKA inhibition might represent a novel therapeutic strategy for OSCC [27]. AURKA was also clearly identified as a hub gene in OSCC by bioinformatics analysis, and in prognostic analysis, AURKA expression level was positively correlated with poor prognosis of OSCC [28,29,30].

Since AURKA is a predictor of chemotherapy response and prognosis for OSCC patients, the binding activity of APS to AURKA was simulated using a molecular docking method. The results showed (Figure S3) that APS can bind to the docking pocket and the binding energy is about −6.03 ± 0.51 kcal/mol, indicating that the component has a good affinity with the target protein. Thus, it can be speculated that APS may be able to bind stably and spontaneously with the corresponding action target AURKA and achieve the therapeutic effect through acting on AURKA receptor.

#### 3.4.2. In Vitro Analysis

Network pharmacology analysis suggested that APS targeted therapy for OSCC was closely related to autophagy. Therefore, in this study, we investigated the effect of autophagy inhibitors, CQ or 3-MA, on MTNs-APS@HA-PNIPAAM hydrogel-induced reduction in cell migration and downregulation of mitochondrial membrane potential.

**Figure 10:**
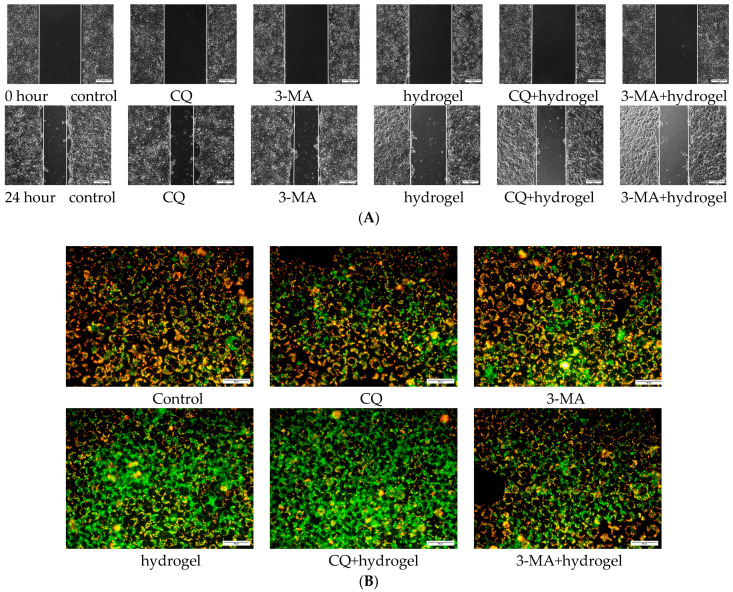
CQ and 3-MA attenuates the inhibitory effect of MTNs-APS@HA-PNIPAAM hydrogels by activating autophagy of CAL-27 cells. The autophagy inhibitors, CQ or 3-MA, were added for 1 h before the addition of composite hydrogel with 200 μg/mL APS. (**A**) Scratching assay; (**B**) JC-1 staining.

The results of scratch test showed that the autophagy inhibitors effectively blocked the inhibitory effect of MTNs-APS@HA-PNIPAAM hydrogel on CAL-27 cell migration. As shown in Figure 10A, the initial scratch area of each group before cell migration was about 771,975 ± 11,222 pixels^2^ with no significant difference. After 24 h of incubation, the control group had a scratch area of 437,482 ± 8467 pixels^2^, the CQ + APS 200 μg/mL group 521,835 ± 23,481 pixels^2^, and the 3-MA + APS 200 μg/mL group 512,920 ± 16,996 pixels^2^. The migratory activity of CAL-27 cells was significantly inhibited under the intervention of autophagy inhibitors, which further indicated that MTNs-APS@HA-PNIPAAM hydrogel may exert its inhibitory effect by activating autophagy in CAL-27 cells.

The autophagy inhibitors were also used to further explore the role of autophagy in MTNs-APS@HA-PNIPAAM hydrogel downregulated mitochondrial membrane potential. The results, as shown in Figure 10B, demonstrated that after 24 h of incubation, the control group exhibited more red fluorescence and less green fluorescence. Upon the addition of MTNs-APS@HA-PNIPAAM hydrogel, a significant increase in green fluorescence and a decrease in red fluorescence were observed. When MTNs-APS@HA-PNIPAAM hydrogel was co-incubated with the autophagy inhibitor CQ, the intracellular green fluorescence was significantly enhanced compared to the hydrogel group. Conversely, co-incubation with the autophagy inhibitor 3-MA resulted in a significant reduction in intracellular green fluorescence. This is due to the fact that CQ, as an autophagy inhibitor, can only partially inhibit the fusion of autophagosomes with lysosomes and subsequent autophagic lysosomal degradation, while 3-MA can block the formation of autophagosomes.

## 4. Discussion

Astragalus polysaccharide has shown great potential in the treatment of OSCC. However, there are certain challenges associated with the application of APS in OSCC treatment. One major concern is the lack of controlled and sustained release, which requires further research and development of appropriate drug delivery systems or strategies. Additionally, APS lacks sufficient targeting ability, leading to limited accumulation in OSCC cells and reduced efficacy. Improving targeting capabilities through targeted drug delivery systems or conjugation with targeting molecules could enhance its therapeutic benefits.

Mesoporous materials, particularly MTNs, have gained significant attention in drug delivery and sustained release applications [31]. These materials possess a well-defined porous structure with interconnected channels, offering a large surface area and high pore volume, which are highly advantageous for drug loading. In this study, MTNs synthesized through the solvothermal method exhibited a specific surface area of 147.059 m^2^/g with a pore size of 3.256 nm. Under transmission electron microscopy, it displayed a characteristic worm-like mesoporous structure. The experimental results demonstrate the successful fabrication of mesoporous titanium dioxide with a well-defined porous structure and desirable surface properties. The high specific surface area suggests a large number of accessible sites for drug loading, enabling efficient encapsulation of therapeutic agents. The worm-like mesoporous structure further enhances the drug loading capabilities.

The integration of MTNs with externally triggered stimuli, including temperature, light, radiofrequency magnetic fields, and ultrasound, to trigger stimulus-responsive drug release, constitutes a rational strategy [32,33]. For example, the pH-sensitive anionic hydrogels with TiO_2_ particles were used for photocatalytic degradation of methylene blue [34]. In this study, MTNs and PNIPAAM hydrogels were combined to offer controlled release properties. The hydrogel can act as a barrier, preventing the burst release of drugs from the mesoporous structure and enabling sustained and controlled drug release over time. PNIPAAM hydrogels can be further modified to exhibit stimuli-responsive behavior, such as temperature sensitivity. Below LCST of PNIPAAM (around 32 °C), the hydrogels retain a swollen state, facilitating drug loading. However, when the temperature exceeds the LCST, the hydrogels undergo a phase transition, collapsing and releasing the loaded drugs in a controlled manner. This unique property allows for triggered drug release upon external stimuli, such as body temperature.

Based on the experimental results, the composite hydrogel demonstrates a minor burst release phenomenon within the first 3 h. Subsequently, a sustained and slow drug release is observed. This indicates that the composite hydrogel achieves sustained and controlled drug release over a specific time frame, effectively prolonging the drug’s therapeutic effect. Furthermore, the composite hydrogel plays a significant role in temperature-responsive release. At room temperature, the composite hydrogel remains in a sol state and takes 24 h to reach swelling equilibrium. At 37 degrees Celsius, the composite hydrogel transitions into a gel state and reaches swelling equilibrium in just 12 h. This suggests that the composite hydrogel is highly sensitive to temperature changes and facilitates controlled drug release under different temperature conditions, whether within the body or outside. When the temperature exceeds a specific threshold (e.g., body temperature), the composite hydrogel undergoes a gel-sol phase transition, triggering drug release.

Additionally, composite hydrogel has shown excellent anti-proliferative and anti-migratory effects on CAL-27 cells. In the MTT assay, the results showed that the composite hydrogel exerted a notable inhibitory effect on CAL-27 cell proliferation. Compared to the control group, the absorbance values of the composite hydrogel-treated group were significantly lower, indicating reduced metabolic activity and cell viability. In the scratch assay, the width of the scratch gap in the composite hydrogel-treated group remained relatively wider compared to the control group, indicating impaired migration ability of the CAL-27 cells. These findings indicate that the composite hydrogel may have potential as a therapeutic agent for suppressing the proliferation and migration of CAL-27 cells, which are important characteristics related to tumor growth and metastasis. Further studies are warranted to investigate the underlying mechanisms and evaluate the potential applications of the composite hydrogel in cancer treatment.

The network pharmacology analysis indicates that the inhibitory mechanism of APS on CAL-27 cell proliferation and migration is associated with cellular autophagy. Since mitochondrial membrane potential reflects early-stage cellular autophagy, we utilized the JC-1 staining method to detect cell autophagy, and the results revealed that as the concentration of APS in the composite hydrogel increased, the green fluorescence intensity gradually increased, suggesting a decrease in mitochondrial membrane potential and the induction of cellular autophagy. The experimental findings align with those of Du et al. [35], suggesting that APS inhibits the proliferation of tumor cell through promoting autophagy. This implies that targeting cellular autophagy could serve as a promising avenue for augmenting the sensitivity of anticancer drugs.

Cellular autophagy is known to be involved in the degradation and recycling of damaged organelles and proteins, playing a crucial role in maintaining cellular homeostasis. Autophagy can help eliminate cancer cells by selectively removing dysfunctional cellular components, preventing the accumulation of damaged molecules, and limiting genomic instability. Furthermore, autophagy can enhance the efficacy of certain anticancer treatments by promoting cell death or sensitizing tumor cells to the cytotoxic effects of drugs. Additionally, autophagy modulation may impact the immune response against tumors, influencing the infiltration and activity of immune cells within the tumor microenvironment. The decrease in mitochondrial membrane potential observed in our experiment suggests that APS induces cellular autophagy in CAL-27 cells when incorporated into the composite hydrogel. The induction of autophagy by APS may contribute to its inhibitory effects on CAL-27 cell proliferation and migration. To investigate the role of cellular autophagy in the migration inhibition, we conducted interventions using autophagy inhibitors CQ and 3-MA. The results demonstrated that the autophagy inhibitors effectively reversed the inhibitory effect of the composite hydrogel on CAL-27 migration. Further research is needed to better understand the intricate mechanisms underlying autophagy in different tumor contexts and to develop therapeutic approaches that effectively harness its beneficial effects while minimizing its detrimental consequences.

## 5. Conclusions

In this study, MTNs-APS@HA-PNIPAAM hydrogels prepared by electron beam irradiation were used as a drug delivery system for the treatment of OSCC. The model drug APS was released slowly from the composite hydrogel and inhibited the proliferation and migration of CAL-27 cells, accompanied by a decrease in mitochondrial membrane potential. The autophagy inhibitors, CQ and 3-MA, blocked the inhibitory effect of composite hydrogel further, confirming that autophagy plays a pivotal role in facilitating the hydrogel’s anticancer activity. It enriches and complements the application of MTNs-loaded hydrogels and irradiation technique in the active targeting of natural drugs to tumor cells, with very important scientific significance. Beyond cancer therapy, the MTNs-NIPAAM hydrogel’s biocompatibility and tunable properties could find applications in tissue engineering, wound healing, and regenerative medicine. The insights gained from this study lay the groundwork for a range of exciting future investigations that could have a transformative impact on the field of oncology and beyond. Moreover, the network pharmacology approach was adopted to predict the pharmacological effects of the model drug APS, and speculate the molecular mechanism of treatment, which will guide the direction of subsequent experimental research. This will further complement the medicinal value of APS in OSCC and provide data support for the development of APS-related pharmaceutical products.

Recently, photocatalytic hydrogels in cancer therapy have been receiving increasing interest. They have been widely used in cancer research for photothermal and photodynamic effects. In the future, hydrogels with multi-modal combined effects such as chemotherapy and photodynamic therapy will be applied, and researchers should work together to ensure the safety and efficiency of hydrogel delivery systems.

## Data Availability

Not applicable.

## Supplementary Materials

The following supporting information can be downloaded at: https://www.mdpi.com/article/10.3390/polym15183659/s1. Figure S1: The differential genes of OSCC. Figure S2: GO enrichment and KEGG pathway analysis represented in a chord plot. Figure S3: Molecular docking models of astragalus polysaccharide and AURKA.
