# Supplementary material for: Mesoporous Titanium Dioxide Nanoparticles—Poly(N-isopropylacrylamide) Hydrogel Prepared by Electron Beam Irradiation Inhibits the Proliferation and Migration of Oral Squamous Cell Carcinoma Cells
Source: Polymers (Basel). 2023 Sep 5;15(18):3659. doi: 10.3390/polym15183659 (PMC10535267; doi:10.3390/polym15183659)
Supplement: Supplementary file 1 [file polymers-15-03659-s001.zip › polymers-2560378-supplementary.pdf]

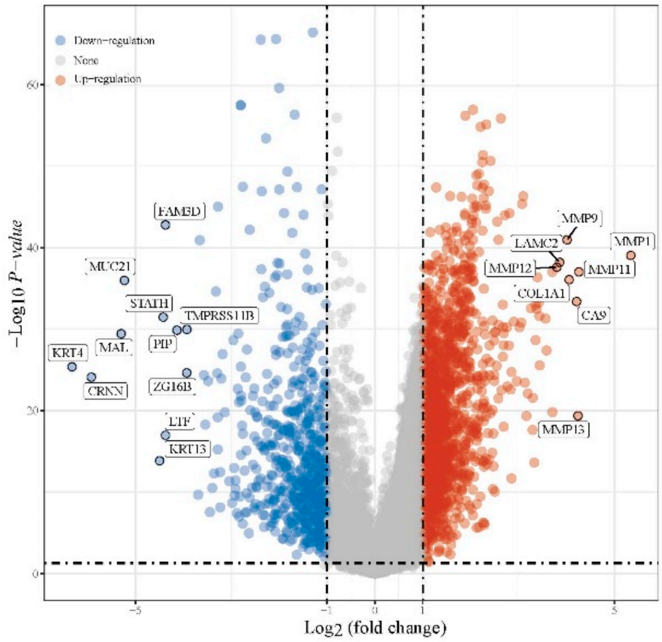

Figure S1:The differential genes of OSCC. Blue: down-regulated genes, Red: up-regulated genes, gray: undifferentially expressed genes.

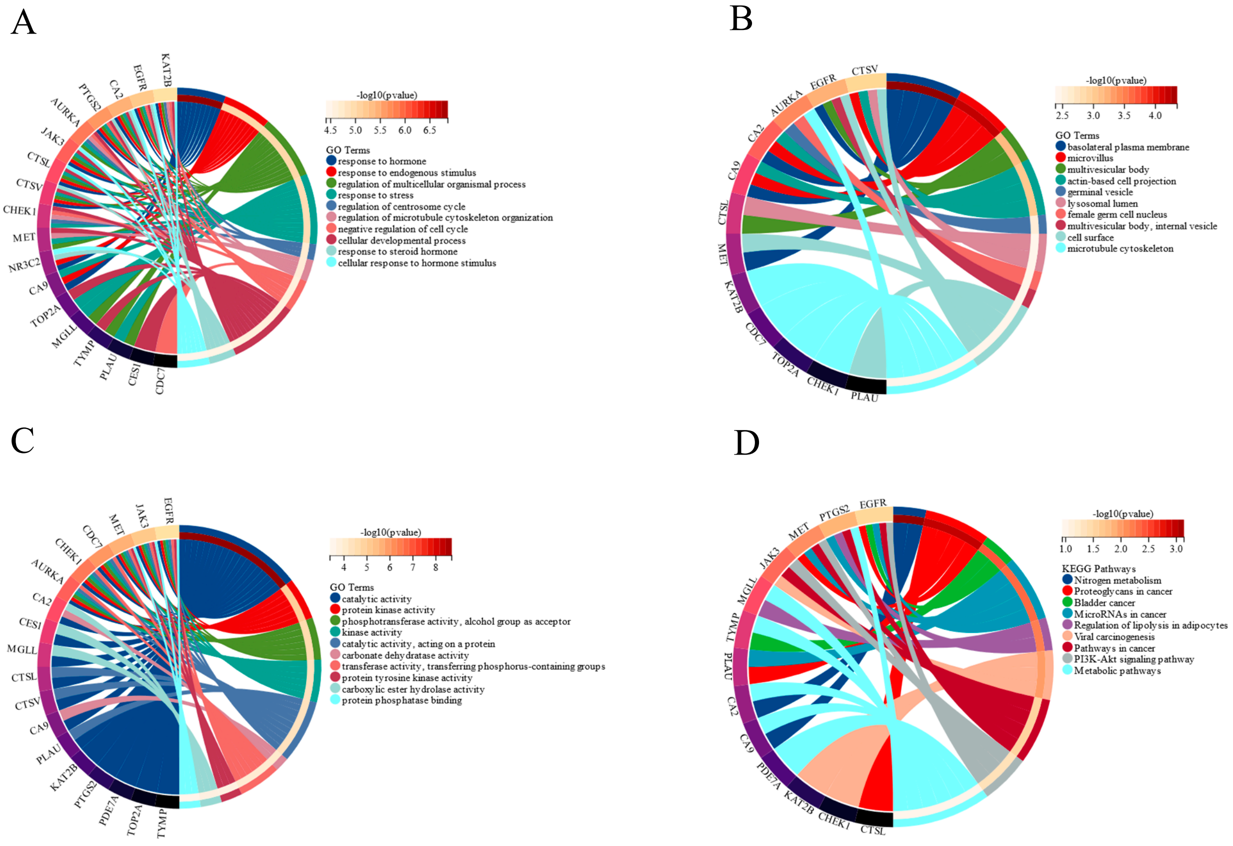

Figure S2. GO enrichment and KEGG pathway analysis represented in a chord plot. A: biological processes; B: cellular components; C: molecular functions; D: signaling pathways.

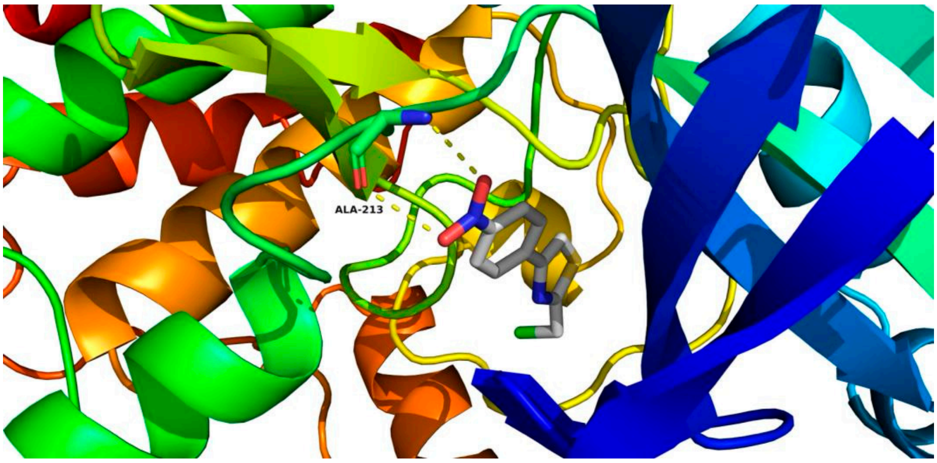

Figure S3. Molecular docking models of astragalus polysaccharide and AURKA.
The binding energy is -6.03±0.51 kcal/mol.
